# The Fish Collagen Supplementation and Proteomic Features in Healthy Women—A Crossover Study

**DOI:** 10.3390/nu17193052

**Published:** 2025-09-24

**Authors:** Marta Stelmach-Mardas, Eliza Matuszewska-Mach, Krzysztof Kustra, Dagmara Pietkiewicz, Jan Matysiak, Dorota Hojan-Jezierska, Marcin Mardas, Leszek Kubisz

**Affiliations:** 1 Department of Obesity Treatment, Metabolic Disorders and Clinical Dietetics, Poznan University of Medical Sciences, 60-355 Poznan, Poland; 2 Department of Inorganic and Analytical Chemistry, Poznan University of Medical Sciences, 60-806 Poznan, Poland; eliza.matuszewska@ump.edu.pl (E.M.-M.); dagmarapietkiewicz3@gmail.com (D.P.); jmatysiak@ump.edu.pl (J.M.); 3 Department of Biophysics, Poznan University of Medical Sciences, 60-780 Poznan, Poland; krzysztofkustra@interia.pl (K.K.); djeziers@ump.edu.pl (D.H.-J.); lkubisz@ump.edu.pl (L.K.); 4 Department of Gynaecological Oncology, Institute of Oncology, Poznan University of Medical Sciences, 60-569 Poznan, Poland; marcin.mardas@ump.edu.pl

**Keywords:** fish collagen, silver carp, supplementation, proteome, diet

## Abstract

**Background**: Using fish collagen supplements in daily nutrition may positively influence health and healthy aging. However, their systemic, molecular-level effects on humans are not well characterized. Therefore, given the scarcity of proteomic data, this study aimed to assess the serum proteomic changes during the fish collagen supplementation in healthy women. **Methods**: This was a crossover interventional study. Thirty healthy women received either 5 mL of fish gel collagen (from silver carp: *Hypophthalmichthys molitrix*) supplementation with 200 mL of pure water for 40 days or 200 mL of pure water for 40 days only. The washout between the fish collagen and pure water supplementation was 40 days. The nutritional status and dietary intake were assessed. Proteome analyses were conducted using a MALDI-TOF mass spectrometer in a positive linear mode in the *m*/*z* 1000–10,000 range. **Results**: The diet of the women in this study was not well-balanced. Supplementation did not affect nutritional status. Only water content significantly increased. During the fish collagen supplementation, the following discriminative proteins were identified: Filamin-A, Filamin-B, actin, Vimentin, Tropomyosin beta chain, 40S ribosomal protein S8, ATP-dependent RNA helicase DHX8, and FERM domain-containing protein 4A. **Conclusions**: Changes in serum proteins may reflect broader cytoskeletal remodeling and cellular adaptation resulting from collagen intake.

## 1. Introduction

The skin of silver carp (*Hypophthalmichthys molitrix*) is one of the sources of active collagen and fish collagen peptide extraction that can be used for novel product development in the field of cosmetology or nutritional supplements [1,2]. The biological activity depends on peptide preparation and composition [3]. Products/extracts currently available on the market may contain plenty of small proteins and are characterized by native collagen, which positively influences the expression of endogenous collagen in fibroblasts and bone regeneration [2]. It has been shown that fish collagen has a positive effect on delaying skin aging by interfering with aging parameters and compensating for oxidative damage and physiological loss in human fibroblasts [4]. Additionally, collagen preparations may be useful in wound healing and support skin repair by inducing the expression of CC and CXC chemokines and the potent angiogenic factor VEGF-A in individuals [5]. A recent study provided insight into the dynamic oxidative modifications of collagen peptides, which were shown to be associated with changes in their bioactivities [6].

A major role in collagen synthesis is played by absorbed amino acids, which may act as building materials for collagen production in fibroblasts, and the oligopeptides, which as ligands adhere to the surface of fibroblasts and stimulate the secretion of collagen, elastin, and hyaluronate [4]. It was recently suggested that a fraction of the total amount of collagen is synthesized and removed on a daily basis without being incorporated into the lifelong permanent collagen [7]. From a clinical point of view, hyperglycemia and insulin play a role in the progression of fibrosis in patients with nonalcoholic steatohepatitis through the upregulation of connective tissue growth factor [8]. It has also been shown that insulin and IGF-1, which stimulate hepatic stellate cell mitogenesis and collagen synthesis, may act in concert to promote liver fibrosis in vivo by a differential activation of PI3-K- and ERK1-dependent pathways [9].

Despite this growing body of evidence, there is a significant gap in the knowledge regarding the effects of fish collagen on humans. The majority of existing studies of collagen supplementation measured only secondary endpoints, such as skin hydration and elasticity, dermal density, and facial wrinkle reduction [10,11,12,13]. However, data on the direct molecular-level effects of ingested fish collagen are lacking. Proteomics analyses offer professionals a universal tool for protein detection in plasma, which may help to optimize the dose and the form of collagen supplementation and, therefore, give insight into how to improve effectiveness. It was indicated that resistance exercise training in combination with collagen peptide supplementation may improve the percentage of fat free mass and muscle strength [14]. Women of reproductive age who invest in so-called healthy aging constitute a significant portion of people using collagen preparations.

The aim of this study was to assess the serum proteomic changes during the fish collagen supplementation in healthy women.

## 2. Materials and Methods

### 2.1. Study Design

This was a crossover interventional study. This study was approved by the Bioethics Committee of the Poznan University of Medical Sciences, No.: DWL II/0172/19; 7 December 2018. This study was carried out in accordance with the Declaration of Helsinki. Women included in this study provided written consent to participate after reading the study protocol. Blood samples were collected from volunteers at the outpatient clinic.

### 2.2. Study Population

Thirty healthy volunteers who signed informed written consent were recruited according to the following inclusion and exclusion criteria. The inclusion criteria included the following:

- Female;
- Caucasian race;
- Body Mass Index (BMI) value ranging between 18.5 kg/m^2^ and 29.9 kg/m^2^;
- Age between 18 and 45;
- No physical complaints in the month preceding this study.

The exclusion criteria were as follows:

- Diagnosed diet-related diseases: diabetes, hypercholesterolemia, obesity, hypertension, endocrine disorders, liver diseases, and autoimmune diseases;
- Acute diseases;
- Cancer in the last 5 years;
- Taking hypoglycemic, lipid-lowering, hypertensive, and psychotropic drugs, which are pharmacotherapeutics that might affect digestion and absorption of protein;
- Antibiotic therapy within the last month;
- Taking dietary supplements and beverages that include collagen in the last 3 months;
- Pregnancy;
- Breastfeeding;
- Lactation;
- Smoking.

### 2.3. Methods

#### 2.3.1. Nutritional Status and Skin Elasticity Assessment

The nutritional status was assessed using BMI, the waist-to-hip ratio (WHR), and body fat percentage. Body weight and body height were measured using a medical scale with an altimeter (Radwag, Radom, Poland), taken in a standing position, without shoes, and in light clothes with an accuracy of 0.1 kg and 0.5 cm, respectively. Waist circumference measurement was made in the middle of the distance between the lower edge of the costal arch and the upper crest of the iliac bone using an anthropometric measure with an accuracy of 0.1 cm. Hip circumference was measured at the height of the upper greater trochanters of the femurs using an anthropometric measure with an accuracy of 0.1 cm. Body composition was assessed using the Tanita BC-601 (Tanita Europe BV, Amsterdam, The Netherlands). The Cutometer Dual MPA 580 (Koeln, Germany) was used to assess the skin elasticity (R7). The Cutometer’s operating principle was based on the suction of the skin, which is made possible by the pressure (450 mbar) generated into the aperture. The aperture into which the skin is sucked has a diameter of 2 mm. The skin was drawn into the aperture for a duration of 2 s. After that, the suction was released, and a 2-second relaxation phase occurred (3 times).

#### 2.3.2. Dietary Intervention

This study utilized a randomized, crossover design. Participants were randomly allocated into one of the two groups using a computer-generated randomization list:

- Group 1 received fish collagen supplementation for 40 days, followed by a 40-day washout period, then water for 40 days.
- Group 2 received water for 40 days, followed by a 40-day washout period, then fish collagen supplementation for 40 days.

**Figure 1:**
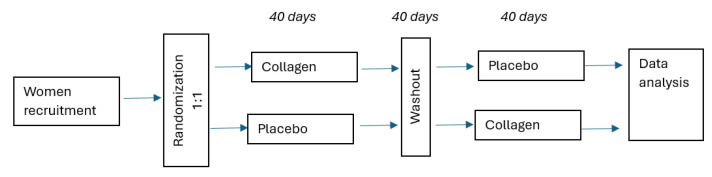
Flowchart.

Firstly, participants assigned to Group 1 received 5 mL of fish gel collagen (from silver carp; Lorenz & Woźniak General Partnership, Poland) supplementation with 200 mL of pure water for 40 days administered directly into the oral cavity, whereas participants in Group 2 (the control group) received only 200 mL of pure water for 40 days. The volunteers took the supplement with water once a day in the morning. A 40-day washout period was then implemented in both groups. After the washout, participants crossed over: those who had received collagen plus water switched to water only (control), and those who had received water only switched to collagen plus water. All individuals included in this study had internal self-control (Figure 1). Monitoring the collagen supplement intake was carried out regularly directly by telephone. Additionally, the assessment of the women’s diet was carried out with the use of a 3-day nutritional interview, which included 2 working days and 1 weekend day (Dietetyk, Poznan, Poland).

#### 2.3.3. The Amino Acid Composition of Collagen

The detailed amino acid composition of the collagen supplement (Lorenz & Woźniak General Partnership, Poland) was determined at the Chemical Laboratory of the Faculty of Animal Nutrition and Feed Management, Poznań University of Life Sciences, using an automatic AAA T-339 amino acid analyzer (Mikrotechna Praha Co., Prague, Czech Republic) (Table 1).

#### 2.3.4. Serum Sample Pretreatment

Blood samples were taken in the follicular phase of the menstrual cycle in the morning, fasting. Venous blood samples were collected into serum tubes with clotting activator: S-Monovette^®^ Serum Gel CAT, 4.9 mL, cap brown, (LxØ): 90 × 13 mm (Sarstedt, Nümbrecht, Germany). The samples were then centrifuged at 2500× *g* for 10 min, and the serum was collected. Before proteomic analysis, each serum sample was prepared using ZipTip C18 solid-phase extraction tips according to the manufacturer’s protocol (Millipore, Bedford, MA, USA) to remove salts, lipids, and other contaminants, as well as excess high-abundance proteins. This procedure reduces the masking of low-abundance proteins by high-abundance proteins. Each sample was diluted in 0.1% trifluoroacetic acid (TFA) in water (1:5 ratio) to obtain a 10 µL sample volume for further analysis. ZipTip C18 tips were conditioned with acetonitrile (ACN) and 0.1% TFA. Aspirate–dispense cycles of the entire sample were performed for binding peptides to the equilibrated ZipTip pipette tips. After the washing step with 0.1% TFA, the bound peptides were eluted with 4 µL of 50% ACN with 0.1% TFA.

#### 2.3.5. MALDI-TOF Proteomic Profiling

The proteomic analysis of eluates from the ZipTip C18 serum pre-treatment (Section 2.3.4) was performed at the Department of Inorganic and Analytical Chemistry, Poznan University of Medical Sciences. This analysis aims to identify changes in the low-abundance serum proteins and those resulting from collagen supplementation. Samples were prepared for MALDI-ToF analysis as follows: The matrix solution consisted of α-cyano-4-hydroxycinnamic acid (HCCA) prepared at 0.3 g/L in a 2:1 (*v*/*v*) mixture of ethanol and acetone. For analysis, each eluate was combined with the matrix solution in a 1:10 volume-to-volume ratio (eluate/matrix). One microliter of this sample and matrix solution mixture was spotted onto the AnchorChip Standard 800 m target plate (Bruker Daltonics, Bremen, Germany) and left to crystallize at room temperature. Each sample was analyzed in three technical replicates. MS data were acquired using an UltrafleXtreme MALDI-TOF/TOF instrument (Bruker Daltonics, Bremen, Germany) controlled by flexControl (v3.4, Bruker Daltonics, Bremen, Germany). The measurements were performed in the positive linear mode with 2000 laser shots per sample in the *m*/*z* 1000–10,000 range. The mass spectrometer calibration was performed with a mixture of a Protein Calibration Standard I and a Peptide Calibration Standard (commercial products from Bruker Daltonics, Bremen, Germany) combined in a 5:1 volumetric ratio (protein standard to peptide standard). The average mass deviation was less than 100 ppm. The matrix suppression mass cut-off was set at 700 Da. The mass spectrometer parameters were as follows: pulsed ion extraction at 260 ns and lens at 6.4 kV.

#### 2.3.6. NanoLC-MALDI-TOF/TOF MS Discriminative Peaks Identification

Identification of discriminative proteins and peptides was conducted using a nanoLC-MALDI-TOF/TOF MS system [14,15]. Serum samples underwent pretreatment with ZipTip C18 micropipette tips before nanoLC separation. The nanoLC setup included an EASY-nLC II nanoflow HPLC system (Bruker Daltonics, Bremen, Germany) and a Proteineer-fc II fraction collector (Bruker Daltonics, Bremen, Germany). Nanosystem components consisted of an NS-MP-10 BioSphere C18 trap column (20 mm × 100 μm I.D., particle size of 5 μm, and pore size of 120 Å) and an Acclaim PepMap 100 column (150 mm × 75 μm I.D., particle size of 3 μm, and pore size of 100 Å). A gradient elution method of 2–50% ACN in 96 min (mobile phase A: 0.05% TFA in water; mobile phase B: 0.05% TFA in 90% ACN) was employed, with a flow rate set at 300 nL/min and a 4 μL sample eluent injection volume. The eluate from the nanoLC separation was collected as 384 automated fractions (75 nL each). Each fraction was mixed with 420 µL of a matrix solution before being spotted onto an AnchorChip Standard MALDI target plate. The matrix solution was prepared by combining four components. To a base solvent of 90:10 (*v*/*v*) acetonitrile and 0.1% aqueous TFA, a saturated HCCA stock solution (also in the base solvent), 10% aqueous TFA, and 100 mM ammonium bicarbonate were added to achieve a final volumetric ratio of 748:36:8:8, respectively. The matrix solution was thus prepared for a final concentration of 0.2% TFA and 1 mM ammonium bicarbonate.

The nanoLC system operation was managed using HyStar 3.2 software. The MS experiments were conducted using an UltrafleXtreme mass spectrometer (Bruker Daltonics, Bremen, Germany) operated by FlexControl 3.4, working in reflector mode within the *m*/*z* 700–3500 range. External calibration utilized a mixture of Peptide Calibration Standard II (Bruker Daltonics, Bremen, Germany). The standard was prepared by reconstituting the lyophilized commercial peptide mixture in 125 µL of a solution containing 30% acetonitrile and 0.1% trifluoroacetic acid (TFA) in water. This matrix consisted of a base solvent of 85% ACN/0.1% TFA, a saturated stock of α-cyano-4-hydroxycinnamic acid (HCCA) in 90% ACN/0.1% TFA, 10% aqueous TFA, and 100 mM ammonium bicarbonate, combined in a volumetric ratio of approximately 748:36:8:8, respectively. The final concentration of TFA was 0.2%, and ammonium bicarbonate was 1 mM.

For analysis, the reconstituted peptide standard was mixed with this dedicated matrix solution at a 1:200 volumetric ratio (*v*/*v*, standard-to-matrix ratio). A volume of 420 nL of the final mixture was spotted onto each designated calibration position on the target plate.

During the MALDI-TOF analysis, precursor ion selection was facilitated by WARP-LC software 2.1. Data collection, processing, and evaluation were performed using FlexAnalysis 3.4 and BioTools 3.2 software. Discriminative proteins and peptides were identified based on a SwissProt database using the Mascot 2.4.1 search engine, with taxonomic restriction to Homo sapiens, and specific search parameters were set as follows: fragment ion mass tolerance of *m*/*z* ± 0.7, precursor ion mass tolerance of ± 50 ppm, peptide charge of +1, and monoisotopic mass.

#### 2.3.7. Statistical Analysis

MS spectra were analyzed in the mass range from 1 to 10 kDa. The spectra grouping function was applied to group all analyzed sample replicates into one biological replicate. Further steps were processed upon one averaged spectrum per sample. Each spectrum was normalized to the total ion current (TIC). The top hat operator was used for baseline subtraction, with a minimum baseline width set to 10%. The average spectra were smoothed using a Savitzky–Golay filter. The signal-to-noise threshold was set to 5.00. The spectra were recalibrated using the following parameters: 1000 ppm maximum peak shift and 30% match with calibrator peaks. In order to compare the studied groups to create discriminant models, a genetic algorithm (GA) was used [16,17]. GA is based on an evolutionary process, which allows the selection of the most important variables. For the GA, parameters of cross-validation and recognition capability were calculated. The results of this analysis were cross-validated using the “leave one out” method. In order to perform external validation, the group of samples analyzed was divided into two subgroups. In this study, 40 samples (20 from individuals before and 20 from individuals after collagen supplementation) were randomly selected as the “learning” set, while 20 samples (20 from individuals before and 20 from individuals after collagen supplementation) were randomly selected as the “test” set. The correctly classified part of valid spectra [%] was calculated. According to the obtained results, the peaks were depicted for further identification. Results were expressed as medians and percentages. The statistical significance of differences between the collagen group and the control group was determined with the use of the Wilcoxon rank-sum test. The level of significance was set at *p* < 0.05. Statistical analysis was performed in STATISTICA 10.0 (StatSoft Inc., Tulsa, OK, USA) and ClinProTools 3.0 (Bruker Daltonics, Bremen, Germany).

## 3. Results

The amino acid composition of fish collagen was presented in Table 1. Supplementation with fish collagen did not affect the nutritional status of the women, taking into account % fat mass (FM), BMI, or WHR, except for the percentage of water content, which increased significantly (Table 2). An increase in the R7 parameter was visible during the fish collagen supplementation (collagen group: 0.82 ± 0.07 vs. 0.84 ± 0.05; *p* = 0.105; control group: 0.84 ± 0.06 vs. 0.82 ± 0.07; *p* = 0.130). The higher the value of R7, the greater the skin elasticity.

The energy value of the diet was lower than the recommended level [18]. The proportions of protein, fat, and carbohydrate intake indicate the lack of a properly balanced diet. The diet was also characterized by a low supply of dietary fiber. Dietary cholesterol intake did not exceed the recommended value of 300 mg/day (Table 3).

During the proteome analyses, several discriminative proteins between the study groups before and after fish collagen supplementation were identified (Table 4), with only one neuronal cell adhesion molecule differentiating the control group before and after water intake (Table 5). Both isoforms of Filamin (a, b). A complete list of peaks classified as differentiators, including unidentified peaks, is provided in Supplementary Materials (Tables S1 and S2).

**Figure 2:**
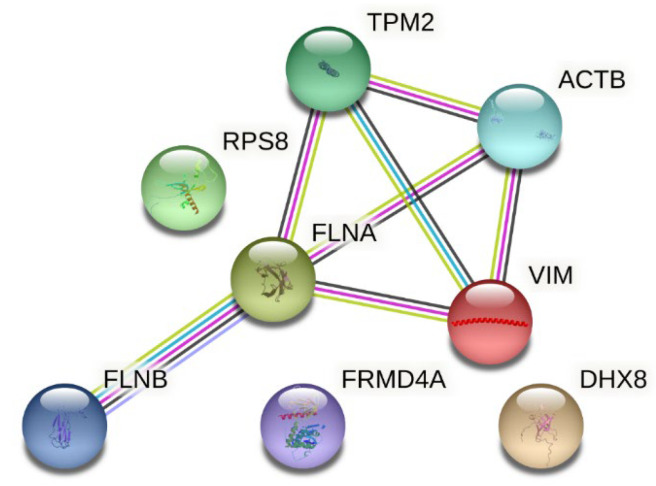
Interactions between proteins differentiating the study group before and after collagen supplementation (source: https://string-db.org (accessed on 5 March 2025)). Legend: TPM2—Tropomyosin beta chain; ACTB—actin, cytoplasmic 1; RPS8—40S ribosomal protein S8; FLNA—Filamin-A; VIM—Vimentin; FLNB—Filamin-B; FRMD4A—FERM domain-containing protein 4A; DHX8—ATP-dependent RNA helicase DHX8. The nodes represent query proteins and the first shell of interactors; inside the nodes, a known or predicted 3D structure is presented. Interactions (represented by lines): known interactions: blue—from curated databases; pink—experimentally determined; predicted interactions: green—gene neighborhood; red—gene fusion; navy—gene co-occurrence; and others: light green—text mining; black—co-expression; and light violet—protein homology.

Selected interactions between proteins differentiating study groups before and after collagen supplementation are presented in Figure 2. STRING analysis shows interactions between molecules and the pathways in which they are involved. As shown in Figure 2, most of the proteins identified as differentiators in this study are related, apart from 40S ribosomal protein S8, FERM domain-containing protein 4A, and ATP-dependent RNA helicase DHX8.

Samples from control individuals—taking water instead of collagen—were also subjected to proteomic analysis. The differential protein identified is shown in Table 5. A complete list of peaks classified as differentiators, including unidentified peaks, is provided in Supplementary Materials (Tables S3 and S4).

## 4. Discussion

The use of fish collagen supplementation in healthy women indicated several protein molecules that build a bridge between molecular research and understanding the role of fish collagen in human nutrition and healthy aging. Selected proteomic research has already contributed to this study on the pathogenesis of diet-related diseases, giving the potential for new biomarker discovery. Nevertheless, the knowledge coming from healthy individuals gives us potential for further proteome investigation and targeted analyses in selected pathways. Changes in serum proteins may reflect broader cytoskeletal remodeling and cellular adaptation resulting from collagen intake.

A recently published meta-analysis [19] indicated that gut peptides are decreased by an increased high-fat, high-carbohydrate diet and by decreased chewing. Nevertheless, it should be highlighted that the study population of women did not follow a specific diet. No dietary restriction was applied over the study time period. The imbalance in the proportion of basic nutrients in the diet, the underestimation of the caloric value of the diet, and the low level of dietary fiber in the diet are part of the nutritional characteristics of the average Pole. As was pointed out by Riberio et al. [19], there is no statistically significant difference in gut peptides between individuals with obesity and leanness in a fasting state. However, the release of gut peptides is affected in individuals with obesity following external stimuli, for example, chewing. Similarly, nutritional research conducted in specific groups of patients may give us a broadened perspective on specific interactions. The decline of collagen begins between the ages of 18 and 29, and after the age of 40, collagen production drops by approximately 1% per year. Importantly, collagen decline can also be increased by factors including excessive sun exposure, smoking, excess alcohol, or lack of sleep. Among identified proteins in the discriminative study group during fish collagen supplementation, two isoforms of Filamin were indicated. Filamin A (FLNa), as an actin-binding protein, is involved in the formation of the cytoskeleton and the regulation of cell adhesion and migration. It plays a role in cell proliferation or differentiation and signal transduction and participates in pseudopodia formation, vesicle transport, or tumor resistance [20]. FLN cross-links cortical actin into a dynamic three-dimensional structure and interacts with a large number of cellular proteins [21,22]. Kao et al. [23] hypothesized that hydrostatic pressure (HP) could upregulate FLN expression mediated by p38 phosphorylation, along with the upregulation of transforming growth factor β (TGF-β), and consequently promote cell migration. It has also been reported that mutations in Filamins A and B may cause a wide range of human diseases [21]. Another interesting protein molecule that was indicated in the current study was Vimentin. Regulation of Vimentin is highly complex and is driven by posttranslational modifications such as phosphorylation and cleavage by intracellular proteases [22]. Dave and Bayless [21] highlighted a pathway involving growth factor-mediated calpain activation, Vimentin cleavage, and MT1-MMP membrane translocation that is required for endothelial cell invasion in 3D environments. As was suggested, this pathway may also regulate the analogous processes of neurite extension and tumor cell invasion. It is a type III intermediate filament (IF) protein, currently one of the most well-known proteins in the IF family that reflects assembly into major cytoskeletal systems in cells of mesenchymal and ectodermal origin [23]. The accumulation of fibroblasts leads to excessive collagen deposition and matrix remodeling, which distort the tissue architecture and contribute to the progressive decline in organ function [24]. The use of Vimentin in targeted pharmacotherapy may help in so-called “healthy wound repair” instead of fibrosis [23]. In general, alpha-smooth muscle actin (alpha-SMA), expressed by activated C-terminal procollagen alpha-1 propeptide, are described in the literature as early markers of fibrogenesis [25]. Actin is functionally associated with Tropomyosin and troponins. F-actin forms a complex with Tropomyosin (TM), a regulatory fibrillar protein (70 kDa) consisting of two intertwined α-helices. TM takes part in the regulation of muscle contraction. It was indicated that early phases of coronary atherosclerosis at the stage of stable atherosclerotic plaques show increased amounts of cytoskeletal proteins, i.e., Tropomyosin β chain, actin, and Vimentin [26]. 40S ribosomal protein S8 (RPS8) is another identified protein in our study. It is primarily located in cytoplasmic messenger ribonucleoprotein granules containing untranslated mRNAs and expressed in all organs and tissues in humans [27,28]. Taking into account the research conducted by Bi et al. [28], we can suspect that RPS8 may be a novel biomarker for the diagnosis of patients with alcohol-associated hepatocellular carcinoma (HCC) as the gene set enrichment analysis (GSEA) demonstrated that RNA polymerase and ribosome pathways were enriched in alcohol-associated HCC samples where RPS8 was highly expressed. FRMD4A was also a protein found to be differentiated during the fish collagen supplementation. It has been shown that FRMD4 is a human epidermal stem cell marker implicated previously in epithelial polarity that is upregulated in squamous cell carcinoma [29]. Martiskainen et al. [30] results suggest that the expression of, i.e., FRMD4A is altered in relation to increasing Alzheimer’s disease (AD)-related neurofibrillary pathology, and that FRMD4A may play a role in amyloidogenic and tau-related pathways in AD.

In order to further interpret the biological significance of the discriminative proteins, we performed a pathway enrichment analysis using the PANTHER classification system (https://pantherdb.org (accessed on 5 March 2025)). The analysis showed that the depicted proteins are annotated in pathways including integrin signaling, cytoskeletal regulation by Rho GTPase, and other signaling cascades, e.g., dopamine receptor and cadherin signaling pathways (Table 6). These findings suggest that the identified proteins are primarily involved in cytoskeletal organization and broader cellular processes such as structural support, intracellular signaling, and cellular adaptation.

Interestingly, no direct enrichment for canonical fibrogenesis or extracellular matrix (ECM) pathways was observed. The explanation could be that the observed proteomic changes caused by collagen supplementation may reflect broader cytoskeletal remodeling and cellular adaptation, rather than a specific activation of pathways related to fibrogenesis. Many of the depicted proteins, such as actin, Vimentin, and Filamins, are multifunctional and play roles in a variety of cellular processes beyond fibrogenesis, including cell migration, wound repair, and tissue remodeling.

**Figure 3:**
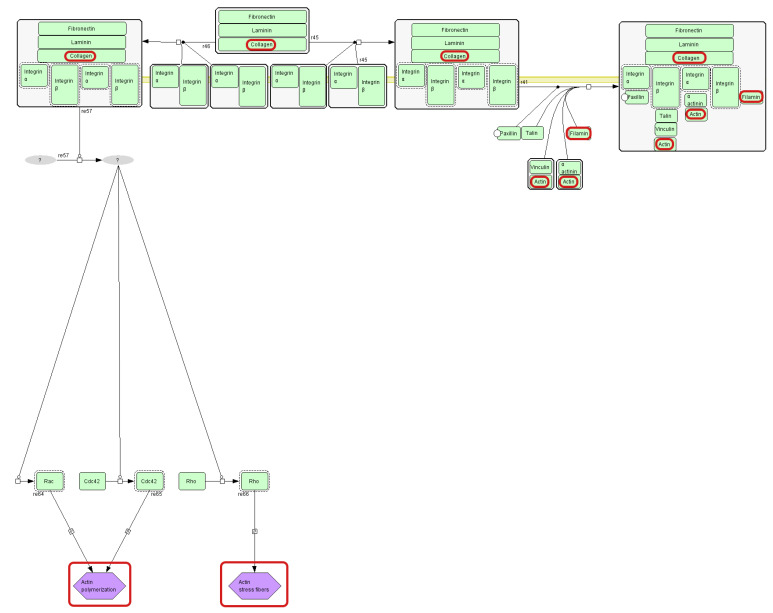
Fragment of integrin signaling pathway diagram (source: https://pantherdb.org). Collagen and actin (protein identified in this study) are marked with red circles.

Filamin-A, Filamin-B, and actin are involved in the integrin signaling pathway. Integrin signaling is initiated when cell-surface integrins engage extracellular matrix (ECM) ligands, leading to actin cytoskeleton remodeling and activation of mitogen-activated protein kinase (MAPK) and other downstream pathways (Figure 3) [31]. According to the available literature, integrins modulate various pathways controlling cellular survival, growth, lineage specification, motility, and metabolic programs [32]. Collagen, one of the ECM ligands, causes the activation of the integrin signaling pathway, leading to, i.e., actin polymerization. This mechanism may account for the altered levels of actin and Filamin observed in our study. However, integrins also function as negative regulators of collagen synthesis [32].

**Figure 4:**
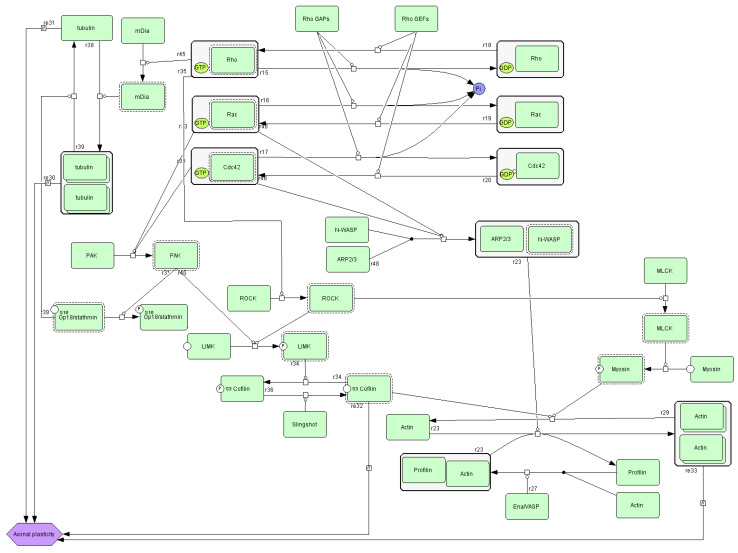
A diagram of cytoskeletal regulation by the Rho GTPase pathway (source: https://pantherdb.org).

Beyond its role in integrin signaling, actin participates in multiple cellular pathways, including cytoskeletal regulation by Rho GTPase (Figure 4). Actin is a major cytoskeletal element of most cells [33]. This protein enables essential processes such as cell relocation and changing shape. These functions require continuous actin network remodeling: filaments are dynamically assembled and disassembled to facilitate shape changes and directional movement. The Rho GTPase family is a central regulatory switchboard for this cytoskeletal reorganization. In vivo, cell migration occurs within complex three-dimensional (3D) extracellular matrices and often along aligned collagen fibers. Recent advances, supported by innovative experimental approaches, have revealed that Rho GTPases exhibit context-specific regulation and functional specialization during migration in 3D matrices, along fibrillar collagen tracks, and in living tissues [34].

This study’s limitations include, on the one hand, aspects of its design; on the other hand, the crossover design clearly demonstrates that each participant serves as their own control in the intervention study.

First, the sample size was limited. Although the crossover design enhances statistical power by using each participant as their own control, this study may still be underpowered to detect small but biologically relevant proteomic changes. Furthermore, the short intervention period of 40 days limits our ability to assess the long-term sustainability of the observed effects. Although the study group was homogeneous, the focus on females of reproductive age gives us the potential for results extrapolation only in this population group. This study was not blinded, which is also a limitation of this study. However, the population and supplementation taken were strictly controlled by the research group. Another limiting factor was compliance monitoring, which was conducted solely via telephone calls, which relies on self-reports only and may overestimate real adherence. However, the crossover design and the controlled supplementation protocol partially mitigated the risk of noncompliance. Moreover, dietary assessment was based on three days and was not combined with a food frequency questionnaire. Furthermore, due to the relatively short (40 days) washout period, potential carry-over effects cannot be entirely excluded. Since this study did not include a validation step for the identified proteins, the findings should be interpreted as preliminary. MALDI-TOF MS is suitable for relative comparisons of proteomic features across samples [35]. Nevertheless, larger cohorts will be needed to validate these candidate markers and fully characterize the observed differences between groups. To address this study’s limitations, subsequent efforts could incorporate quantitative methods, such as ELISA tests, to confirm the proteomic changes caused by the fish collagen supplementation. Searching for further opportunities to investigate the role of fish collagen supplementation in the selected group of oncological patients, i.e., hepatocellular carcinoma or neurodegenerative disorders such as Alzheimer’s disease, seems to be justified.

## 5. Conclusions

Fish collagen supplementation was associated with modulation of specific protein molecules involved in fibrogenesis and physiological wound healing processes. Moreover, changes in serum proteins may reflect broader cytoskeletal remodeling and cellular adaptation resulting from collagen intake. These findings may support further research into the use of fish-derived collagen in both preventive nutrition and potential therapeutic applications.

## Figures and Tables

**Table 1:** The amino acid composition of fish collagen.

Total protein content (%)	65.66
Amino acid (g per 100 g protein)	
Asparagine	6.64
Threonine	2.84
Serine	3.44
Glutamic acid	10.61
Proline	13.55
Cysteine	-
Glycine	21.83
Alanine	10.54
Valine	2.27
Methionine	2.25
Isoleucine	1.57
leucine	2.95
Tyrosine	1.32
Phenylalanine	3.03
Histidine	1.21
Lysine	3.38
Arginine	7.56
Hydroxyproline	5.01

**Table 2:** Characteristics of women taking a fish collagen supplementation (*n* = 30).

Analyzed Parameter		Mean	SD	Median	*p*-Value
Body Weight [kg]	Before	66.3	7.2	66.3	0.8990
	After	66.3	7.3	66.5	
Body Heigh [cm]	Before	168.3	4.66	168	-
Fat Mass [%]	Before	29.8	5.3	29.7	0.5629
	After	31.2	9.9	29.6	
Fat Free Mass [kg]	Before	43.8	3.7	44.2	0.1761
	After	43.8	3.7	44.3	
Bone Mass [kg]	Before	2.33	0.2	2.5	0.8531
	After	2.33	0.2	2.5	
Visceral Fat Mass [kg]	Before	3.9	1.5	4.0	0.9121
	After	3.9	1.5	4.0	
Body Mass Index [kg/m^2^]	Before	23.4	2.1	23.3	0.9838
	After	23.4	2.2	23.0	
Water Content [%]	Before	51.4	4.1	52.1	<0.0001
	After	52.1	4.2	52.8	
Hip Circumference [cm]	Before	97.8	9.2	97.5	0.1664
	After	97.9	9.3	97.5	
Waist Circumference [cm]	Before	89.1	7.5	89.0	0.2011
	After	89.3	7.4	89.3	
Waist-to-Hip Ratio	Before	0.91	0.82	0.87	0.0867
	After	0.91	0.80	0.88	
Waist-to-Heigh Ratio	Before	0.53	0.04	0.52	0.1512
	After	0.53	0.04	0.52	

**Table 3:** The energy value of the diet and the supply of selected nutrients in the study population (*n* = 30).

Analyzed Parameter	Mean	SD	% of Recommended Intake
Energy [kcal]	1863	353	84.7
Protein [g]	65.9	7.8	110.9
Fat [g]	36.5	8	89.5
Cholesterol [mg]	272.4	62.1	<300
Carbohydrates [g]	318	33.8	115.9
Dietary fiber [g]	19.7	4.9	65.7
Calcium [mg]	557	93	55.7
Magnesium [mg]	196	61	62.3
Iron [mg]	7	1	48.0
Vitamin A [μg]	488	65	69.7
Vitamin C [mg]	53	13	70.5
Vitamin E [mg]	5	1	67.5

**Table 4:** List of identified proteins discriminative between study groups before and after fish collagen supplementation.

Precursor Ion *m*/*z*	*p*-Value of Wilcoxon Test	Sequence	Accession	Protein Name	Protein Expression in Individuals Before vs. After Supplementation
1506.38	<0.000001	K.ISSLLEEQFQQGK.L	RS8_HUMAN	40S ribosomal protein S8	↓
1099.38	0.0188	K.GTVEPQLEAR.G	FLNA_HUMAN	Filamin-A	↓
1821.60	<0.000001	M.DDDIAALVVDNGSGMCK.A	ACTB_HUMAN	Actin, cytoplasmic 1	↓
1283.44	<0.000001	K.VTVLFAGQHIAK.S	FLNA_HUMAN	Filamin-A	↓
1061.01	<0.000001	K.DMLAALKSR.Q	FRM4A_HUMAN	FERM domain-containing protein 4A	↓
1488.31	<0.000001	K.ATDAEADVASLNRR.I	TPM2_HUMAN	Tropomyosin beta chain	↓
1750.66	0.00000233	R.LQDEIQNMKEEMAR.H	VIME_HUMAN	Vimentin	↑
1450.69	<0.000001	R.EAEMDSIPMGLNK.H	DHX8_HUMAN	ATP-dependent RNA helicase DHX8	↑
1655.67	0.000262	R.GAGGQGKLDVTILSPSR.K	FLNB_HUMAN	Filamin-B	↓
1020.95	0.000079	K.DMLAALKSR.Q	FRM4A_HUMAN	FERM domain-containing protein 4A	↓

The arrows indicate the direction of intensity change in the precursor ions recorded in the MS spectra of serum samples obtained from individuals before and after collagen supplementation. It should be noted that the MALDI-TOF mass spectrometer does not allow quantitative analysis, so the direction of intensity changes should be treated as informative and not directly correlated with protein concentrations.

**Table 5:** Identified protein discriminative between the control group before vs. after water intake.

Precursor Ion *m*/*z*	*p*-Value of Wilcoxon Test	Sequence	Accession	Protein Name	Protein Expression in Individuals Before vs. After Water Intake
1538.54	0.00515	K.LGMAKNEVHLEIK.D	NRCAM_HUMAN	Neuronal cell adhesion molecule	↓

The arrow indicates the direction of intensity change in the precursor ion recorded in the MS spectra of serum samples obtained from individuals before and after water intake. It should be noted that the MALDI-TOF mass spectrometer does not allow quantitative analysis, so the direction of intensity change should be treated as informative and not directly correlated with protein concentration.

**Table 6:** Functional annotation of differentiating proteins identified in this study (source: https://pantherdb.org).

Protein Name	Biological Process	Molecular Function	Pathway
40S ribosomal protein S8	Cellular and metabolic processes	Structural molecule activity	Not assigned
Filamin-A	Not assigned	Not assigned	Dopamine receptor mediated signaling pathway, Integrin signaling pathway, Nicotine pharmacodynamics pathway
Actin, cytoplasmic 1	Cellular process, Developmental process, Multicellular organismal process	Binding, Structural molecule activity	Alzheimer’s disease–presenilin pathway, Cadherin signaling pathway, Cytoskeletal regulation by Rho GTPase, Huntington disease, Inflammation mediated by chemokine and cytokine signaling pathway, Integrin signaling pathway, Nicotinic acetylcholine receptor signaling pathway, Wnt signaling pathway
FERM domain-containing protein 4A	Not assigned	Not assigned	Not assigned
Tropomyosin beta chain	Cellular process, Multicellular organismal process	Binding	Not assigned
Vimentin	Cellular process	Structural molecule activity	Not assigned
ATP-dependent RNA helicase DHX8	Cellular and metabolic processes	ATP-dependent activity, Binding, catalytic activity	Not assigned
Filamin-B	Not assigned	Not assigned	Integrin signaling pathway
Neuronal cell adhesion molecule	Brain development Cell–cell adhesion Axon guidance	Cell–cell adhesion mediator activity	Not assigned

## Data Availability

The original contributions presented in this study are included in the article and Supplementary Materials. Further inquiries can be directed to the corresponding author.

## Supplementary Materials

The following supporting information can be downloaded at: https://www.mdpi.com/article/10.3390/nu17193052/s1, Table S1: Values of chemometric parameters for the applied mathematical Genetic Algorithm (GA) used for statistical analysis; Table S2: Integration regions used for classification based on Genetic Algorithm (GA); Table S3: Values of chemometric parameters for the applied mathematical Genetic Algorithm (GA) used for statistical analysis; Table S4: Integration regions used for classification based on Genetic Algorithm (GA).
