# Supplementary material for: The Fish Collagen Supplementation and Proteomic Features in Healthy Women—A Crossover Study
Source: Nutrients. 2025 Sep 24;17(19):3052. doi: 10.3390/nu17193052 (PMC12525885; doi:10.3390/nu17193052)
Supplement: Supplementary file 1 [file nutrients-17-03052-s001.zip › nutrients-3723857-supplementary.pdf]

Supplementary material

Results

I. Study group before vs after collagen supplementation (n=30)

Table S1. Values of chemometric parameters for the applied mathematical Genetic Algorithm (GA) used for statistical analysis

Cross validation [%]	Recognition capability [%]	External Validation – correct classified part of valid spectra [%]	
		Study group before collagen supplementation	Study group after collagen supplementation
100	100	100	100

Table S2. Integration regions used for classification based on Genetic Algorithm (GA)

Precursor ion m/z	p-Value of Wilcoxon test	Peak area/intensity of class: Study group before collagen supplementation	Peak area/intensity of class: Study group after collagen supplementation	Sequence	Accession	Protein name
**1506.38**	< 0.000001	28.25	5.35	K.ISSLLEEQFQQGK.L	RS8_HUMAN	40S ribosomal protein S8
**1099.38**	0.0188	11.44	7.29	K.GTVEPQLEAR.G	FLNA_HUMAN	Filamin-A
**2988.85**	< 0.000001	1.28	0.75	x	x	x
**4322.32**	< 0.000001	0.63	0.17	m/z > 3500	m/z > 3500	m/z > 3500
**1821.60**	< 0.000001	6.33	2.51	M.DDDIAALVVDNGSGMCK.A	ACTB_HUMAN	Actin, cytoplasmic 1
**4250.98**	< 0.000001	0.54	0.19	m/z > 3500	m/z > 3500	m/z > 3500
**1283.44**	< 0.000001	16.85	6.66	K.VTVLFAGQHIAK.S	FLNA_HUMAN	Filamin-A

**5974.34**	< 0.000001	0.14	0.05	m/x > 3500	m/x > 3500	m/x > 3500
**1061.01**	< 0.000001	27.14	8.57	K.DMLAALKSR.Q	FRM4A_HUMAN	FERM domain-containing protein 4A
**5920.83**	< 0.000001	0.17	0.08	m/z > 3500	m/z > 3500	m/z > 3500
**1488.31**	< 0.000001	15.29	9.94	K.ATDAEADVASLNRR.I	TPM2_HUMAN	Tropomyosin beta chain
**1750.66**	0.00000233	23.54	38.16	R.LQDEIQNMKEEMAR.H	VIME_HUMAN	Vimentin
**1450.69**	< 0.000001	3.01	6.32	R.EAEMDSIPMGLNK.H	DHX8_HUMAN	ATP-dependent RNA helicase DHX8
**1655.67**	0.000262	8.77	5.67	R.GAGGQGKLDVTILSPSR.K	FLNB_HUMAN	Filamin-B
**1020.95**	0.000079	11.92	6.56	K.DMLAALKSR.Q	FRM4A_HUMAN	FERM domain-containing protein 4A

x – precursor ion not identified

m/z > 3500 - precursor ion not identified due to limitations of the method

II. Control group before vs after water intake (n=30)

Table S3. Values of chemometric parameters for the applied mathematical Genetic Algorithm (GA) used for statistical analysis

**Cross validation [%]**	**Recognition capability [%]**	**External Validation – correct classified part of valid spectra [%]**	
		**Placebo group before water intake**	**Placebo group after water intake**
85.48	96.67	90	77.3

Table S4. Integration regions used for classification based on Genetic Algorithm (GA)

**Precursor ion m/z**	**p-Value of Wilcoxon test**	**Peak area/intensity of class: Placebo I**	**Peak area/intensity of class: Placebo II**	**Sequence**	**Accession**	**Protein name**
**7769.05**	0.615	0.08	0.08	m/z > 3500	m/z > 3500	m/z > 3500

**1538.54**	0.00515	9.12	4.56	K.LGMAKNEVHLEIK.D	NRCAM_HUMAN	Neuronal cell adhesion molecule
**5920.71**	< 0.000001	0.56	0.11	m/z > 3500	m/z > 3500	m/z > 3500
**3365.47**	0.136	0.43	0.35	x	x	x
**2604.93**	0.771	1.46	1.33	x	x	x
**1467.36**	0.267	157.71	137.29	x	x	x
**3194.95**	0.000028	5.36	3.06	x	x	x
**1506.53**	0.000228	6.8	4.45	x	x	x
**9428.03**	0.0128	0.12	0.06	m/z > 3500	m/z > 3500	m/z > 3500
**3159.98**	0.0387	2.58	1.67	x	x	x
**1099.82**	0.391	7.09	8.54	x	x	x
**1546.78**	0.467	13.55	14.73	x	x	x
**3211.14**	0.000819	0.86	0.59	x	x	x
**3974.44**	0.0332	2.29	1.39	m/z > 3500	m/z > 3500	m/z > 3500
**5987.59**	0.0167	0.08	0.06	m/z > 3500	m/z > 3500	m/z > 3500

x – precursor ion not identified

m/z > 3500 - precursor ion not identified due to limitations of the method
